# Direct evidence for a bystander effect of ionizing radiation in primary human fibroblasts

**DOI:** 10.1054/bjoc.2000.1665

**Published:** 2001-03

**Authors:** O V Belyakov, A M Malcolmson, M Folkard, K M Prise, B D Michael

**Affiliations:** Gray Laboratory Cancer Research Trust, Mount Vernon Hospital, PO Box 100,Northwood, Middlesex, HA6 2JR, UK

**Keywords:** ionizing radiation, bystander effect, primary fibroblast, micronucleus

## Abstract

Bystander responses underlie some of the current efforts to develop gene therapy approaches for cancer treatment. Similarly, they may have a role in strategies to treat tumours with targeted radioisotopes. In this study we show direct evidence for the production of a radiation-induced bystander response in primary human fibroblasts. We utilize a novel approach of using a charged-particle microbeam, which allows individual cells within a population to be selected and targeted with counted charged particles. Individual primary human fibroblasts within a population of 600–800 cells were targeted with between 1 and 15 helium ions (effectively, α-particles). The charged particles were delivered through the centre of the nucleus with an accuracy of ± 2 μm and a detection and counting efficiency of greater than 99%. When scored 3 days later, even though only a single cell had been targeted, typically an additional 80–100 damaged cells were observed in the surviving population of about 5000 cells. The yield of damaged cells was independent of the number of charged particles delivered to the targeted cell. Similar results of a 2–3-fold increase in the background level of damage present in the population were observed whether 1 or 4 cells were targeted within the dish. Also, when 200 cells within one quadrant of the dish were exposed to radiation, there was a 2–3-fold increase in the damage level in an unexposed quadrant of the dish. This effect was independent of the presence of serum in the culture medium and was only observed when a cell was targeted, but not when only the medium was exposed, confirming that a cell-mediated response is involved. © 2001 Cancer Research Campaign http://www.bjcancer.com


*British Journal of Cancer* (2001) **84**(5), 674–679
© 2001 Cancer Research Campaign
doi: 10.1054/ bjoc.2000.1665, available online at http://www.idealibrary.com on IDEAL

http://www.bjcancer.com

### Direct evidence for a bystander effect of ionizing radiation in primary human fibroblasts

**OV Belyakov, AM Malcolmson, M Folkard, KM Prise and BD Michael**

Gray Laboratory Cancer Research Trust, PO Box 100, Mount Vernon Hospital, Northwood, Middlesex, HA6 2JR, UK

**Summary** Bystander responses underlie some of the current efforts to develop gene therapy approaches for cancer treatment. Similarly, they may have a role in strategies to treat tumours with targeted radioisotopes. In this study we show direct evidence for the production of a radiation-induced bystander response in primary human fibroblasts. We utilize a novel approach of using a charged-particle microbeam, which allows individual cells within a population to be selected and targeted with counted charged particles. Individual primary human fibroblasts within a population of 600–800 cells were targeted with between 1 and 15 helium ions (effectively, α-particles). The charged particles were delivered through the centre of the nucleus with an accuracy of ± 2 μm and a detection and counting efficiency of greater than 99%. When scored 3 days later, even though only a single cell had been targeted, typically an additional 80–100 damaged cells were observed in the surviving population of about 5000 cells. The yield of damaged cells was independent of the number of charged particles delivered to the targeted cell. Similar results of a 2–3-fold increase in the background level of damage present in the population were observed whether 1 or 4 cells were targeted within the dish. Also, when 200 cells within one quadrant of the dish were exposed to radiation, there was a 2–3-fold increase in the damage level in an unexposed quadrant of the dish. This effect was independent of the presence of serum in the culture medium and was only observed when a cell was targeted, but not when only the medium was exposed, confirming that a cell-mediated response is involved. © 2001 Cancer Research Campaign http://www.bjcancer.com

**Keywords:** ionizing radiation; bystander effect; primary fibroblast; micronucleus

The common perception of the pathways involved in radiation effects in cellular systems is that direct damage to nuclear DNA is a requirement which leads to mutation, transformation or cell death only in the initially damaged cell. This view has been challenged with observations of radiation-induced bystander effects. These are measures of changes in cells, which were not initially traversed by the tracks of ionizing radiation, also referred to as non-targeted effects (Little, 2000; Michael et al, 2000; Ward, 2000 for reviews). These observations may have significant implications for current risk estimates for low doses of radiation exposure. The current human respiratory tract model for radiation protection assumes that traversals of α-particles through the nuclei of the target cells within the lung alone leads to the induction of cancer (ICRP, 1994). As bystander effects occur when only a few cells within a population are hit (Nagasawa and Little, 1992) the implications for extrapolation from conventional experimental data, where high doses and high numbers of cells targeted have been used (to that of relevance to radiation risk) need to be addressed. Radiation-induced bystander responses may be different from other chemically mediated bystander responses. For example, bystander responses are criticality important for improving the efficacy of gene therapy approaches for cancer treatment, such as those using the hsvTK-ganciclovir system (Mesnil et al, 1996), where diffusion of known toxic metabolites leads to neighbouring cell killing. However, if radiation-induced bystander responses are significant they may provide a novel mechanism of improving targeted-radiation approaches.

*Received 19 July 2000*
*Revised 13 November 2000*
*Accepted 17 November 2000*

*Correspondence to:* KM Prise

Two different manifestations of a radiation-induced bystander effect have previously been reported. Studies have reported the production of culture medium-derived factors induced by low-LET radiation in epithelial cells (Seymour and Mothersill, 1997; Mothersill and Seymour, 1998; Mothersill et al, 2000) but not in fibroblasts. Other studies monitoring the effects of low doses of α-particles, where only a few cells within a population are traversed have also shown media-derived factors. Two species have been observed (Deshpande et al, 1996; Lehnert and Goodwin, 1997); one which is short-lived and is produced when serum-containing medium is irradiated in the absence of cells and one which is longer lived and requires the irradiation of cells. These studies have suggested that the short-lived factor could be involved in the formation of superoxide radicals, possibly as products of lipid peroxidation. The long-lived cell-dependent factor was postulated to be a cytokine such as TNFα because of its known SCE-inducing activity. Further studies by the group confirmed the involvement of reactive oxygen species (ROS) such as hydrogen peroxide and superoxide anions. Underlying these responses may be an involvement of gap junctional intracellular communication (Azzam et al, 1998; Bishayee et al, 1999), although this has not been observed in all systems (Seymour and Mothersill, 1999). Another important aspect is the dependence on radiation quality or LET (linear energy transfer), particularly at the lowest possible dose, that of a single particle traversal (Prise et al, 1998).

In this study, using a charged particle microbeam system (Folkard et al, 1997a, 1997b), we have tested directly whether bystander effects occur in populations of primary human fibroblasts where individual cells can be located and targeted with precise numbers of charge particles. We show evidence that even when a single cell within a population is traversed with a single helium ion a bystander response is observed.

### MATERIALS AND METHODS

### Cell culture and irradiation

Primary human AG01552B fibroblasts were obtained from the National Institute of Ageing Cell Repository (Coriell Institute for Medical Research, USA). These were routinely maintained at low passage number in α-modified Eagles Minimal Essential Medium (αMEM) supplemented with 20 % (v/v) fetal calf serum, penicillin (100 IU ml$^{-1}$) and streptomycin (100 μg ml$^{-1}$) at 37˚C in an atmosphere of 95% air:5% $CO_2$. For microbeam experiments, plateau phase cells were seeded into specially designed dishes (Folkard et al, 1997a) consisting of a 34 mm diameter base composed of a 4 μm thick polypropylene membrane which had been pretreated with 1 μg ml$^{-1}$ CellTak adhesive (Becton Dickinson, USA). Cells were seeded 16 h prior to irradiation to allow full attachment and >90% were in $G_1$ at the time of irradiation. Typically cells were seeded at a density to allow 600–800 cells per 10 × 10 mm area of the dish. 2 h prior to irradiation cells were incubated with 1 μm Hoechst 33258. At the time of irradiation the cell culture was replaced with fresh medium (including serum) containing 20 mM HEPES and irradiation performed at room temperature. Cells, which were irradiated, had particles delivered through the centre of the cell nucleus. The irradiation procedure typically took around 10 min after which fresh medium was added to the cells and incubation continued at 37˚C for up to 3 days prior to scoring. In some experiments cells were pre-treated with a dose of 240 kV X-rays delivered at a dose rate of 1.8 Gy min$^{-1}$, approximately 10 min before placing them on the microbeam stage. Full details of the physical set-up of the microbeam have been given in previous publications (Folkard et al, 1997a, 1997b). Helium-3 ions with an LET of 100 keV μm$^{-1}$ were used as surrogate α-particles. Due to constraints imposed by the accelerator system, helium-3 ions, rather than α-particles (helium-4), had to be used to achieve adequate penetration of the cells. However, in terms of their biological effectiveness both radiations are considered to be equivalent.

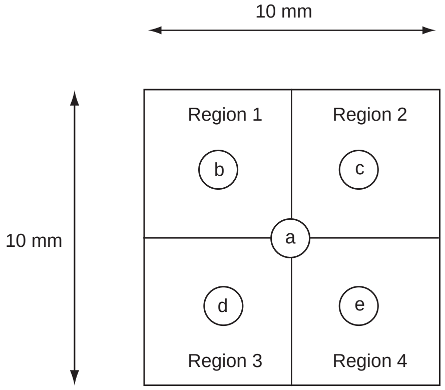

**Figure 1** Layout of areas of dish used for targeted experiments. Cells were found at individual locations (a, b, c, d, or e) and irradiated. Alternatively, all the cells within one quadrant (i.e. regions 1, 2, 3 or 4) were automatically located and revisited with or without irradiation

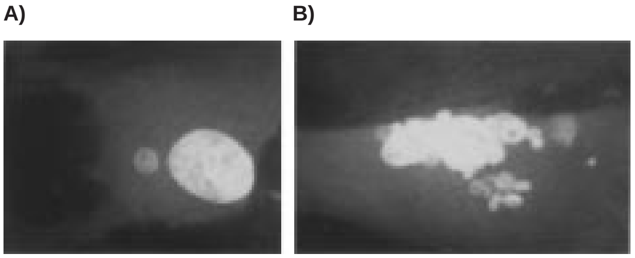

**Figure 2** Images of a damaged cell scored 3 days after irradiation with helium-3 ions. (**A**) shows a single micronucleus within the same cell with a normal nucleus. (**B**) shows heavily damaged nuclei probably within more than one cell with some evidence of fragmentation

### Micronucleus analysis

For scoring of damaged cells, the medium was removed, the monolayer washed in phosphate-buffered saline (PBS), fixed in 100% methanol and stained for 20 min with 0.5% (w/v) acridine orange. This was followed by washing and destaining in PBS for 1 h, followed by air-drying, before scoring. Micronuclei appeared as green coloured round bodies well separated from the main nucleus and the number of cells with micronuclei were determined for each dish as previously described (Belyakov et al, 1999). Some apoptotic cells were also observed during scoring and these were classified on the basis of morphological criteria (Kerr et al, 1972).

### RESULTS

In this study, 3 types of experiment were performed in specially constructed 4 μm polypropylene-based dishes which were divided into 4 quadrants (each 5 × 5 mm) in software (Figure 1). Normally, 600–800 cells were seeded in the whole dish. Dishes were then placed on the micropositioning stage of the microbeam and one of the following routines performed:

1. One cell located in the centre of the area (a) and exposed to 1–15 helium-3 particles.
2. One cell (b, c, d, or e) located in each of the four regions 1, 2, 3, 4 and exposed to 1–15 particles.
3. All the cells in one area (e.g. region 1) found and irradiated. All the cells in a second region located and (e.g. region 2) used as a control.

Figure 2A shows a typically damaged cell, stained with acridine orange, scored 3 days after irradiation with a micronucleus contained within the cytoplasm. Figure 2B shows a heavily fragmented group of nuclei, which typically were around 10% of all the damaged cells, observed in the irradiated experiments. Table 1 shows the distributions of damaged cells scored 3 days after 1 or 4 cells were irradiated in the starting population. 3 days was chosen as the scoring time as this represents the peak formation of micronucleated cells in this population in studies where conventionally delivered (i.e. non-targeted) helium ions were used (Belyakov et al, 1999). Typically, the level of damaged cells in the control dishes was around 1% given absolute numbers of damaged cells of between 30–80 cells, depending on the number of cells scored 3 days later. Targeting a single cell leads on average to 3% damaged cells being detected, typically leading to the observation of around 120–180 damaged cells, 3 days later (see Table 1). This equates to around 100 damaged cells being produced within a population of 5000 cells when a single cells is irradiated. Interestingly, we also

*© 2001 Cancer Research Campaign* 

**Table 1** Numbers of damaged cells scored in control dishes and those where 1 or 4 cells had been targeted with precise numbers of helium ions

| Number of particles per targeted cell | Number of cells targeted per dish | Total cells scored | Total number of damaged cells | Fraction of cells damaged |
|---|---|---|---|---|
| 0 | 1 | 4588 | 48 | 0.010 |
| 0 | 1 | 5153 | 75 | 0.015 |
| 0 | 1 | 6108 | 79 | 0.013 |
| 0 | 1 | 5067 | 82 | 0.016 |
| 0 | 1 | 3891 | 41 | 0.011 |
| 0 | 1 | 6893 | 80 | 0.012 |
| 0 | 4 | 3076 | 30 | 0.010 |
| 0 | 4 | 4536 | 36 | 0.008 |
| 0 | 4 | 3429 | 54 | 0.016 |
| 0 | 4 | 2130 | 16 | 0.008 |
| 0 | 4 | 7428 | 68 | 0.009 |
| 0 | 4 | 5500 | 41 | 0.007 |
| 1 | 1 | 3704 | 126 | 0.034 |
| 1 | 1 | 4591 | 159 | 0.035 |
| 1 | 1 | 6023 | 183 | 0.030 |
| 1 | 1 | 5212 | 115 | 0.022 |
| 1 | 1 | 6102 | 191 | 0.031 |
| 1 | 4 | 3813 | 94 | 0.025 |
| 1 | 4 | 3800 | 115 | 0.030 |
| 3 | 1 | 3562 | 105 | 0.029 |
| 3 | 1 | 4326 | 110 | 0.025 |
| 3 | 4 | 4911 | 165 | 0.034 |
| 5 | 1 | 3324 | 117 | 0.035 |
| 5 | 1 | 5021 | 162 | 0.032 |
| 5 | 1 | 4568 | 170 | 0.037 |
| 5 | 4 | 3256 | 110 | 0.034 |
| 5 | 4 | 3091 | 99 | 0.032 |
| 5 | 4 | 2692 | 67 | 0.025 |
| 5 | 4 | 5240 | 170 | 0.032 |
| 10 | 1 | 4731 | 55 | 0.012 |
| 10 | 4 | 4458 | 127 | 0.028 |
| 10 | 4 | 4402 | 108 | 0.025 |
| 15 | 1 | 4588 | 136 | 0.030 |
| 15 | 1 | 4989 | 140 | 0.028 |
| 15 | 4 | 3944 | 165 | 0.042 |
| 15 | 4 | 3724 | 115 | 0.031 |

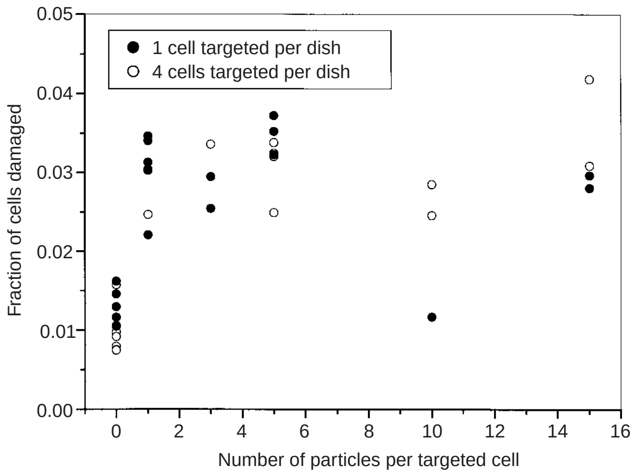

**Figure 3** Fraction of damaged cells measured in the population after a single cell (𝟙) or 4 cells (●) are exposed to individually counted particles. Cells were scored 3 days after irradiation

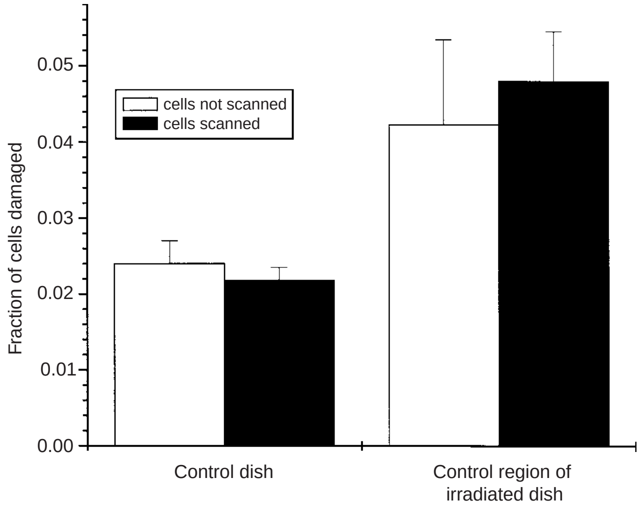

**Figure 4** Level of damaged cells present in the control regions of irradiated dishes versus dishes where no cells were irradiated ($P < 0.05$). Open bars represent dishes where cells were not automatically scanned and revisited using our computerized imaging system observed some heavily damaged cells indicative of apoptotic cells, although at a lower frequency than for micronucleation. In this study however, we have grouped these cells together with the micronucleated ones and classified them as total cell damage (Abend et al, 2000). The control population in these cases was also exposed to particle traversals. In this case a single particle was

 © 2001 Cancer Research Campaign placed in each dish but in an area without a cell present. In some experiments, 1000–2000 particles were delivered in an area of the dish with no cells present as part of the beam alignment beam. Again, no significant increase in the level of damaged cells was observed relative to untargeted dishes. Overall, the total numbers of cells present for scoring 3 days later were not significantly different in dishes where cells had been directly targeted from those where cells had not been directly targeted. From scoring these damaged cell types (Table 1), we obtained a plot of the fraction of damaged cells versus particle number delivered to the selected cell (Figure 3). Importantly, the yields of damaged cells are independent of the numbers of particles delivered giving a constant damaged fraction of around 3%.

We also determined the effect of irradiating 4 cells within the population and measuring the damaged cells. These targeted cells were selected at random towards the centre of each of the 4 quadrants of the dish (see Figure 1). Table 1 also shows the distributions of damaged cells observed under these conditions. Similar to the targeting of a single cell, an increase frequency of damaged cells is observed in the population. Again the increase is independent of the number of particles delivered to the targeted cells (see Figure 3).

In a separate series of experiments we irradiated 25% (150–200) of the cells present with varying doses of helium ions. This we did by automatically finding and locating the cells within 1 quadrant using our computerized imaging system. An increased level of damage was observed in the non-exposed cells, scored in a different quadrant. The level of damaged cells was independent of whether we scanned and located cells at the time of irradiation in the control or irradiated regions (see Figure 4). As for the experiments where we had targeted only 1 or 4 cells we observe a 2-fold increase in the level of damaged cells.

We have also determined whether the response we observe is dependent on the presence of serum in the medium at the time of irradiation, as this has been reported to be a source of short-lived bystander activity (Emerit, 1994; Deshpande et al, 1996; Lehnert and Goodwin, 1997). Figure 5 shows the degree of bystander response in the absence of serum. Also shown is the effect of pre-treating the dish with X-rays (0.1 Gy) prior to irradiating a single cell. Irradiating every cell with 0.1 Gy leads to a significant increase in the level of damaged cells present. Despite this, however when we then target a single cell within this population we still observe a bystander effect leading to an additional ~2-fold increase in the level of damaged cells. We have also tested the ability of the effect to be transferred by addition of media and serum from irradiated cells onto dishes with non-exposed cells. No significant change in the level of damaged cells in a control dish was observed when it had medium transferred to it from a dish in which a single cell had been targeted.

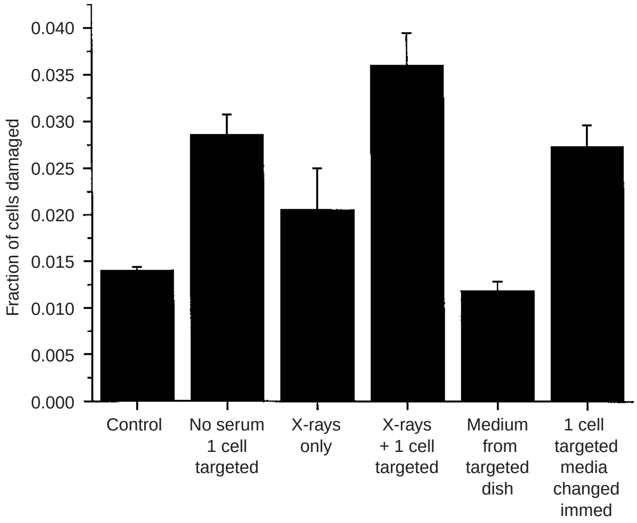

**Figure 5** Modulation of the effects of the bystander response with damaged cells scored 3 days after irradiation. 1) Control dishes where an area was targeted without a cell present. 2) A single cell was targeted with helium ions in the absence of serum ($P < 0.001$). 3) Cells were exposed to 0.1 Gy of X-rays with no cells targeted, in the presence of serum ($P < 0.05$). 4) After exposure to X-rays a single cell was targeted with helium ions, in the presence of serum ($P < 0.001$). 5) Medium (+serum) from dishes where a single cell was targeted was added to unexposed dishes immediately after irradiation and incubated for 30 min (not significant). 6) A single cell was targeted within a dish and the media (+serum) changed immediately after irradiation for fresh medium, (+serum) within 5 minutes ($P < 0.05$). In all these cases no significant difference in the level of effect was observed by changing the number of helium ions (1–15) targeted to the individual cell

### DISCUSSION

In this study we have demonstrated direct evidence for the production of a radiation-induced bystander effect in primary human fibroblasts. Damaged cells were scored 3 days after irradiation as our previous studies in this system have shown that this is the peak expression time for damaged cells in experiments with conventional radiations (Belyakov et al, 1999). Damaged cells were present across the entire area of the dish (10 × 10 mm). However, some evidence for non-uniformity of the expression of the damage (using cluster analysis) was observed i.e. when a damaged cell is present there is an increased probability of a damaged cell being close by. The same phenomena may also be present in the unexposed controls however, and due to the small numbers of damaged cells present it is difficult to rule this out statistically.

In terms of absolute numbers, the targeting of a single cell leads to an additional 100 cells on average being damaged and these can be located throughout the region. Given this level of damage amplification, it should in theory be very easy to inactivate a population of cells when these are exposed to charged particles as the bulk of the effect will be due to a bystander response. In practice, this level of damage amplification is not observed when conventional exposures of average numbers of particles are delivered (Belyakov et al, 1999). Also, if every cell is targeted with a single helium ion, the level of damaged cells present is around 5% (Malcolmson et al, submitted). Our experiments where we pretreated the whole population uniformly with a low dose of X-rays also suggest that the effect is not dependent on the presence of non-hit cells, as a bystander effect is still observed when a single cell is subsequently targeted (Figure 5). The effect of targeting a *single* cell with helium ions under these conditions produces a higher fraction of damaged cells compared to that when *every* cell is targeted with 0.1 Gy of X-rays, indicative of a significant damage amplification.

In this study, we have concentrated on measuring chromosomal damage, in particular micronucleation, which will frequently lead to cell death. Other workers have shown that radiation-induced genomic instability can occur via the bystander effect (Lorimore et al, 1998) leading to both chromosomal and chromatid aberrations. Thus the measurements of only micronucleated cells we have made here may lead to an underestimation of the effects. Increased proliferation has been reported to occur after irradiation

© 2001 Cancer Research Campaign of cell populations with low numbers of α-particles, where only a few cells are traversed by tracks (Iyer and Lehnert, 2000). This occurs via the production of TGFβ and decreased levels of TP53 and CDKN1A. In our study we have not observed any significant changes in cell proliferation within the time window of these experiments. However limited numbers of cells were used in these experiments (see Table 1). We cannot rule out that some of the damaged cells we observe may be due to instability induced in the initially exposed population. Given the modest increase in growth reported by other workers (~1.4 fold at day 3; Iyer and Lehnert, 2000) and the low initial numbers of cells exposed here, it suggests that if instability is significant it is predominantly observed in bystander cells. Evidence for altered mutation frequencies in the surviving progeny of irradiated CHO cells has also recently been reported (Nagasawa and Little, 1999). Overall the bystander effect we report here is small, i.e. a 2–3 fold increase in the background frequency of damage. This, however may still be significant, particularly in terms of risk when one considers low doses of relevance to environmental exposures to ionizing radiation, and it may be of relevance to the development of treatments using targeted radionuclides, where not all cells are successfully targeted but may need to rely on a bystander response (Michael et al, 2000). Also, it is likely that many cells that survive may be altered, for example, being subject to mutational changes (Nagaswa and Little, 1999; Zhou et al, 2000). In these studies a similar effect is observed in terms of a 3–5 fold increase in the expected levels of mutations, which may also depend on the numbers of cells targeted.

No attempt has been made in the present work to determine the mechanisms underlying the bystander response, and we have simply quantified the degree of effect under controlled conditions. Several likely pathways have been reported to be involved. 1) Oxidative stress production leading to increased radical formation, including superoxide and lipid peroxide formation (Narayanan et al, 1997). 2) Cytokine release from irradiated cells (Narayanan et al, 1999). 3) Gap junctional intercellular communication (Azzam et al, 1998). Although the cells used in this study were seeded at low density (~8 per $mm^2$), we cannot rule out the involvement of cell–cell contact-mediated communication. Confocal imaging of these cells shows cytoplasmic projections up to several 100 μm in some cells of the population at the time of irradiation (results not shown). In other studies, we have preliminary evidence for the production of ROS when the fibroblast cells used here are targeted with helium ions delivered to the nucleus, or to the cytoplasm only (Ozols et al, 2000).

The observation of a radiation-induced bystander effect is of significance not just in terms of radiation risk but also in terms of therapeutic use of ionizing radiation. Much of the current interest in therapeutic targeting of radioisotopes is influenced by limitations in the ability to deliver dose uniformly to the tumour volume (Boyd et al, 1999). If bystander responses, in terms of cell killing, can be switched on in neighbouring tumour cells or indeed switched off in surrounding normal cells, potential new approaches may be developed to improve the efficacy of treatments using targeted radioisotopes. They may also be of significance at the relatively low doses used for fractionated therapies, if the general response of no dose-dependence observed in this study and others is important and differences occur between normal and tumour cell responses. Recent studies showing a bystander effect in partially irradiated rat lung confirm the importance of the response and its potential relationship to older data showing abscopal effects (Kahn et al, 1998). Further studies are required to delineate the differences between the response of directly targeted cellular effects and those produced in non-targeted bystander cells.

In summary, we present direct evidence for the production of a radiation-induced bystander response in non-hit cells neighbouring those targeted with individual charged particles through their nucleus. This response is observed when only a single cell within a population is targeted with a single helium ion and is independent of the dose delivered to the targeted cell.

### ACKNOWLEDGEMENTS

The authors are grateful to the Cancer Research Campaign and the Department of Health (UK) for supporting these studies.

### REFERENCES

Abend M, Kehe K, Riedel M and Van Beuningen D (2000) Correlation of micronucleus and apoptosis assays with reproductive cell death can be improved by considering other modes of death. *Int J Radiat Biol* **76**: 249–259

Azzam EI, de Toledo SM, Gooding T and Little JB (1998) Intercellular communication is involved in the bystander regulation of gene expression in human cells exposed to very low fluences of alpha particles. *Radiat Res* **150**: 497–504

Belyakov OV, Prise KM, Mothersill C, Folkard M and Michael BD (2000) Studies of bystander effects in primary urothelial cells using a charged-particle microbeam. *Radiat Res* **153**: 235.

Bishayee A, Rao DV and Howell RW (1999) Evidence for pronounced bystander effects caused by nonuniform distributions of radioactivity using a novel three-dimensional tissue culture model. *Radiat Res* **152**: 88–97

Boyd M, Cunningham SH, Brown MM, Mairs RJ and Wheldon TE (1999) Noradrenaline transporter gene for radiation kill by 131I meta-iodobenzylguanidine. *Gene Ther* **6**: 1147–1152

Deshpande A, Goodwin EH, Bailey SM, Marrone BL and Lehnert BE (1996) Alpha-particle-induced sister chromatid exchange in normal human lung fibroblasts: Evidence for an extranuclear target. *Radiat Res* **145**: 260–267

Emerit I (1994) Reactive oxygen species, chromosome mutation, and cancer: possible role of clastogenic factors in carcinogenesis. *Free Rad Biol Med* **16**: 99–109

Folkard M, Vojnovic B, Hollis KJ, Bowey AG, Watts SJ, Schettino G, Prise KM and Michael BD (1997a) A charged particle microbeam: II A single-particle micro-collimation and detection system. *Int J Radiat Biol* **72**: 387–395

Folkard M, Vojnovic B, Prise KM, Bowey AG, Locke RJ, Schettino G and Michael BD (1997b) A charged-particle microbeam. I. Development of an experimental system for targeting cells individually with counted particles. *Int J Radiat Biol* **72**: 375–385

ICRP: Human respiratory tract model for radiological protection. A report of a Task Group of the International Commission on Radiological Protection. (1994) *Ann ICRP* **24**: 1–482

Iyer R and Lehnert BE (2000) Factors underlying the cell growth-related bystander responses to alpha particles. *Cancer Res* **60**: 1290–1298

Khan MA, Hill RP and Van Dyk J (1998) Partial volume rat lung irradiation: an evaluation of early DNA damage. *Int J Radiat Oncol Biol Phys* **40**: 467–476

Kerr JF, Wyllie AH and Currie AR (1972) Apoptosis: a basic biological phenomenon with wide-ranging implications in tissue kinetics. *Br J Cancer* **26**: 239–257

Lehnert BE and Goodwin EH (1997) Extracellular factor(s) following exposure to α-particles can cause sister chromatid exchanges in normal human cells. *Cancer Res* **57**: 2164–2171

Little JB (2000) Radiation carcinogenesis. *Carcinogenesis* **21**: 397–404

Lorimore SA, Kadhim MA, Pocock DA, Papworth D, Stevens DL, Goodhead DT and Wright EG (1998) Chromosomal instability in the descendants of unirradiated surviving cells after alpha-particle irradiation. *Proc Natl Acad Sci USA* **95**: 5730–5733

Mesnil M, Piccoli C, Tiraby G, Willecke K and Yamasaki H (1996) Bystander killing of cancer cells by herpes simplex virus thymidine kinase gene is mediated by connexins. *Proc Natl Acad Sci USA* **93**: 1831–1835

Michael BD, Belyakov OV, Folkard M, Malcolmson AM, Ozols A and Prise KM (2000) Implications of bystander effects for radiobiology, In: M Moriarty, C Mothersill, C Seymour, M Edington, JF Ward and RJM Fry (Eds) *Radiation Research*, Vol. 2. Allen Press, Lawrence, KS. pp. 397–402

© 2001 Cancer Research Campaign

Mothersill C and Seymour CB (1998) Cell–cell contact during gamma irradiation is not required to induce a bystander effect in normal human keratinocytes: evidence for release during irradiation of a signal controlling survival into the medium. *Radiat Res* **149**: 256–262

Mothersill C, Stamato TD, Perez ML, Cummins R, Mooney R and Seymour CB (2000) Involvement of energy metabolism in the production of 'bystander effects' by radiation. *Br J Cancer* **82**: 1740–1746

Nagasawa H and Little JB (1992) Induction of sister chromatid exchanges by extremely low doses of α-particles. *Cancer Res* **52**: 6394–6396

Nagasawa H and Little JB (1999) Unexpected sensitivity to the induction of mutations by very low doses of alpha-particle radiation: evidence for a bystander effect. *Radiat Res* **152**: 552–557

Narayanan PK, Goodwin EH and Lehnert BE (1997) α-particles initiate biological production of superoxide anions and hydrogen peroxide in human cells. *Cancer Res* **57**: 3963–3971

Narayanan PK, LaRue KE, Goodwin EH and Lehnert BE (1999) Alpha particles induce the production of interleukin-8 by human cells. *Radiat Res* **152**: 57–63

Ozols A, Prise KM, Trott K-R, Folkard M and Michael BD (2000) The role of oxidative stress and DNA damage in genomic instability: targeted microbeam studies in primary human fibroblasts. *Radiat Res* **153**: 229–230

Prise KM, Belyakov OV, Folkard M and Michael BD (1998) Studies of bystander effects in human fibroblasts using a charged particle microbeam. *Int J Radiat Biol* **74**: 793–798

Seymour CB and Mothersill C (1997) Delayed expression of lethal mutations and genomic instability in the progeny of human epithelial cells that survived in a bystander-killing environment. *Radiat Oncol Invest* **5**: 106–110.

Seymour C and Mothersill C (1999) Cell communication and the "bystander effect". *Radiat Res* **151**: 505–506

Ward JF (2000) Non-DNA targeted effects and DNA models, In: M Moriarty, C Mothersill, C Seymour, M Edington, JF Ward and RJM Fry (Eds) *Radiation Research*, Vol. 2. Allen Press, Lawrence, KS. pp. 336–341

Zhou H, Randers-Pehrson G, Waldren CA, Vannais D, Hall EJ and Hei TK (2000) Induction of a bystander mutagenic effect of alpha particles in mammalian cells. *Proc Natl Acad Sci USA* **97**: 2099–2104

*© 2001 Cancer Research Campaign*
